# Comparing spinal anesthesia approaches for transurethral lithotripsy in patients with proximal ureteral stones: A randomized clinical trial of bupivacaine alone *versus* bupivacaine with fentanyl

**DOI:** 10.25122/jml-2023-0109

**Published:** 2023-10

**Authors:** Hossein Khoshrang, Reza Shahrokhi Damavand, Hamidreza Nasseh, Ardalan Akhavan Tavakoli, Samaneh Esmaeili, Mehdi Ghaffari, Maryam Shakiba

**Affiliations:** 1 Anesthesiology Research Center, Alzahra Hospital, School of Medicine, Guilan University of Medical Sciences, Rasht, Iran; 2 Urology Research Center, Razi Hospital, School of Medicine, Guilan University of Medical Sciences, Rasht, Iran; 3 Cardiovascular Diseases Research Center, Heshmat Hospital, School of Medicine, Guilan University of Medical Sciences, Rasht, Iran

**Keywords:** Bupivacaine, fentanyl, anesthesia, spinal, ureteroscopic surgery

## Abstract

Despite the benefits of spinal anesthesia and the preference of anesthesiologists for this technique, it is less accepted by urologists due to the proximity of the stone place in the ureter and the possibility of pain, restlessness, and occasional movements of the patient during surgery. The current study investigated the success of bupivacaine plus intrathecal fentanyl in patients undergoing transurethral lithotripsy (TUL). In this randomized clinical trial, from April 2021 to September 2021, 54 patients with proximal urolithiasis candidates for TUL were enrolled. Patients were randomly categorized into two groups: group A received bupivacaine 10 mg and 0.5 ml of normal saline, while group B received bupivacaine 10 mg with 0.5 ml (25µg) of intrathecal fentanyl. According to our findings, about 74% of the patients were men, and the mean age of the patients was 66.14±22.46 years. The onset time of the sensory block, sensory block level, pain score, degree of relaxation, depth of the motor block, occurrence of anesthesia complications, oxygen saturation, and mean arterial blood pressure were not significantly different between the two groups. However, the duration of the motor block in group B was longer than in group A (p<0.001). Also, retropulsion was observed only in five patients (18.5%) in group A, significantly higher than in group B (p=0.019). Bupivacaine with fentanyl 25µg provided adequate spinal anesthesia with lower retropulsion in patients with urolithiasis who are candidates for TUL.

## INTRODUCTION

Spinal anesthesia (SA) is a safe and straightforward technique that is most commonly used for surgery of the abdominal and lower extremity [1, 2]. It is the first and standard choice for many urological surgeries. It is also commonly used in transurethral surgeries, making it easy to detect the symptoms of overhydration, bladder perforation, and transurethral resection of prostate (TURP) syndrome [3]. There is a tendency to perform transurethral lithotripsy (TUL) with SA due to fewer complications than general anesthesia [2, 4]. Spinal anesthesia is performed with various local anesthetics and neuraxial drugs with different temporal effects [1-2], among which bupivacaine is the most commonly used despite its short duration of action. This has led to bupivacaine needing additional adjuvant drugs to increase its duration of action [5, 6]. Incorporating opioids as anesthetic adjuvants alters block characteristics by stimulating opioid receptors beyond the central nervous system. Additionally, this practice diminishes the requirement for postoperative opioids in intravenous patient-controlled analgesia (IV-PCA). Subsequently, it reduces the possible adverse effects of opioids, such as respiratory depression, nausea, sedation, etc. [7-9]. Fentanyl is a powerful synthetic opioid with a potency of 50 to 100 times greater than morphine. In addition, fast onset of action, simple formulation, ease of production at a high rate, and cost-effectiveness have led to the use of fentanyl in many surgeries [7, 10-12].

Despite the clinical benefits of SA and its widespread use, urologists are unsure about using it in transurethral surgeries due to the possibility of patient movement, restlessness, and subsequent insufficient control over the procedure. However, there is an apparent increase in demand for SA in urological surgeries and even in transurethral procedures because of fewer postoperative side effects. The significance of understanding the pharmacology of new adjunctive drugs in SA underscores the relevance of this study in addressing these concerns. The current investigation evaluated the effects of intrathecal fentanyl in co-administration with bupivacaine during TUL under spinal anesthesia, focusing on patient comfort, surgical success, and possible side effects.

## MATERIAL AND METHODS

### Study design and participants

In this randomized clinical trial conducted between April 2021 and June 2021, patients with proximal ureteral stones less than 15 mm who were candidates for transurethral lithotripsy (TUL) at Razi Educational Hospital in Rasht were prospectively enrolled. Patients aged between 15 and 65 years, of both genders, with ASA Class I/II, were included. Patients were excluded if they had a recent history of opioid analgesic use just before surgery, bleeding disorders, chronic pain conditions, cardiovascular or respiratory diseases, infections at the site of block injection, pregnancy, or a known allergy to bupivacaine and fentanyl. Additionally, individuals requiring supplementary anesthesia due to an unsatisfactory block or those with absolute or partial prohibitions for intrathecal injection were not included. Considering an error protection of 0.05, a power of 0.8, an effect size of 83%, and assuming a dropout rate of 10%, the sample size required per group was 27 to achieve the desired statistical significance for the alternative hypothesis, i.e., adequate spinal anesthesia when adding fentanyl.

### Study procedure and monitoring

Patients were given 30-50 µg/kg midazolam as a sedative before surgery, and all patients received 5 ml/kg normal saline (NS) before intrathecal injection for 15-20 minutes. During surgery, the flow rate was sustained at 4 L/min via a simple oxygen mask. Spinal anesthesia was administered for the patient in a sitting position in the L2–3 vertebral interspace. A 25-gauge Quincke spinal needle was introduced into the dura mater. Patients were randomly divided into two groups by block randomization technique with a 1:1 allocation ratio, a block size of 4. Next, group A (n=27) patients received 2 ml 0.5% bupivacaine (10mg) + 0.5ml of normal saline, and group B (n=27) patients received 2 ml 0.5% bupivacaine (10 mg) + 0.5 ml of fentanyl (25 µg) administered at a rate of 0.1 mL/sec. The injections were carried out ensuring the spinal needle's orifice faced the cephalad and confirming the unimpeded flow of cerebrospinal fluid (CSF). All patients were placed in the supine position immediately after SA and in the lithotomy position.

Patients were provided with 1 mg of midazolam for sedation if necessary during the procedure, sufficient to ensure they could respond to questions. Also, intravenous bolus fentanyl (25µg) was prescribed if analgesia was needed. The patient's blood pressure (BP) was monitored and recorded every 2 to 10 minutes immediately after intrathecal injection and then every 5 minutes until the end of surgery by a non-invasive blood pressure (NIBP) sphygmomanometer. BP was repeatedly measured and recorded inside the recovery ward. In case of moderate arterial hypertension (MAP) below 90 mmHg or less than 80% baseline, 5 mg intravenous ephedrine bolus was repeated, and atropine (0.5 mg) was used for a heart rate of less than 50 beats per minute. Patients in both groups were monitored with continuous electrocardiography (ECG), peripheral oxygen saturation (SpO2), heart rate (HR), and respiratory rate (RR), which were also recorded every five minutes during surgery, at the time of recovery, and admission to the ward.

Sensory block level (SBL) was evaluated in patients with no sense of pain following the pinprick test every minute until reaching the highest level of sensory block. Additionally, the Modified Bromage Scale [13] was used to measure motor block in the lower limbs, with the following scale: 0 = able to lift an extended knee against gravity; 1 = able to flex the knee but not legs; 2 = able to flex toes only; and 3 = inability to move any joints. The onset, peak block level, and sensory and motor block duration were compared between the groups. Patient pain was subjectively measured using a 10-cm visual analog scale (VAS) every 15 minutes during surgery, and the level of sedation was measured by the Ramsay Sedation Scale (RSS). The duration of anesthesia and recovery and the success rate of surgery regarding complete stone removal in both groups were recorded. The frequency and severity of side effects such as shivering, nausea, vomiting, and pruritus were also recorded by direct patient inquiry. The level of sensory block, motor block, and degree of sedation were the primary endpoints. Intra-operative outcomes and postoperative fentanyl and analgesia side effects were the secondary endpoints.

### Statistical analysis

Data were analyzed using the Statistical Package for the Social Sciences version 24.0 (SPSS Inc., Illinois, USA). We used the chi-square and Fisher's exact tests to analyze categorical data. For all other variables, including non-normally distributed data (in accordance with the central limit theorem), we utilized the independent t-test. A p-value<0.05 was considered statistically significant.

## RESULTS

**Figure 1:**
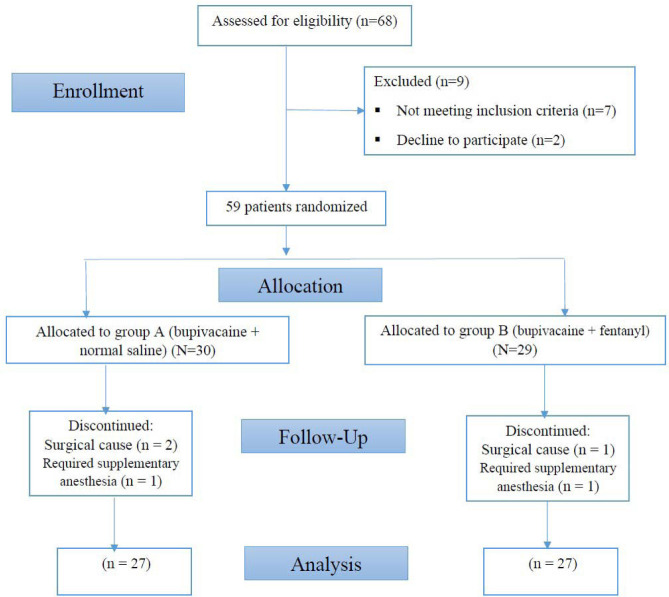
Study flowchart

Fifty-four patients, with a mean age of 46.22±14.7 years, were conveniently divided into two equal groups. The study flowchart is shown in Figure 1. The two groups were comparable regarding gender, age, height, weight, duration of surgery, and ASA status (Table 1). The onset time of sensory block, duration of sensory block, and VAS pain score are shown in Table 2. A significant difference was found in the duration of the sensory block, which was longer in group B (86.11±17.77 min) compared to group A (71.11±11.79 min) (p<0.001). No statistically significant difference was reported between the values of pain based on VAS score in groups A (0.4±1.33) and B (0.11±0.32).

**Table 1:** Demographic characteristics of patients and duration of surgery

Variables		A (Bupivacaine+normal saline)	B (Bupivacaine+Fentanyl)	p-value
**Gender n (%)**	**Male**	19 (47.5)	21 (52.5)	0.535
	**Female**	8 (57.1)	6 (42.9)	
Age* (yrs)		43.44±13.03	49±15.89	0.222
Weight* (kg)		77.44±11.27	83.62±13.55	0.127
Duration of surgery* (min)		50.92±15.69	47.96±13.24	0.457

* Mean±SD

**Table 2:** Comparisons of onset and duration of sensory block and pain score between groups

Variables	A (Bupivacaine+normal saline)	B (Bupivacaine+Fentanyl)	p-value
**Onset time of sensory block (min)**	4.51±1.67	4±1.73	0.124
**Duration of sensory block (min)**	71.11±11.79	86.11±17.77	0.0001
Pain score*	0.4±1.33	0.11±0.32	0.924

* Visual analog scores (VAS)

No significant differences were found in the values of the patient's sedation level based on RSS between groups A and B. None of the subjects in the two groups were able to move their pelvis and knees after receiving SA. The levels of sedation sensory and motor block are summarized in Table 3. No significant intergroup differences were found regarding adverse effects. The most common adverse effect of anesthesia was postoperative shivering in two patients (7.4%) in group A and one (3.7%) in group B. Following that, nausea and vomiting (3.7%) and bradycardia (3.7%) were the most common complications reported only in group B. In terms of surgery complications, the incidence of retropulsion was significantly higher in group A than in group B (18.5% *vs*. 0.0%) (p=0.019). Based on the outcome, the stone-free rate was 100% in group B (n=27) and 81.5% in Group A (n=22). However, no significant difference in residual stones was observed between the two groups after conducting the Fisher's Exact test. There were no statistically significant differences in any mentioned parameters between the two groups (p>0.05). The logistic regression binary analysis, which included all variables in the equation using the Enter method, revealed that none of the variables had a significant impact on retropulsion in the group receiving bupivacaine.

**Table 3:** The levels of sedation, sensory, and motor block

Block	A (Bupivacaine+normal saline)	B (Bupivacaine+Fentanyl)	p-value
**Level of sedation** 1 Patient is anxious and agitated or restless, or both 2 Patient is cooperative, oriented, and tranquil 3 Patient responds to commands only 4 Patient exhibits brisk response to light glabellar tap or loud auditory stimulus 5 Patient exhibits a sluggish response to light glabellar tap or loud auditory stimulus 6 Patient exhibits no response	5 (18.5) 20 (74.1) 1 (3.7) 0 1 (3.7) 0	4 (14.8) 22 (81.5) 1 (3.7) 0 0 0	0.861
**Peak Sensory block level** T4 T6 T8 T10 T12	0 2 (7.4) 4 (14.8) 16 (59.3) 5 (18.5)	0 1 (3.7) 2 (7.4) 19 (70.4) 6 (18.5)	0.374
Motor block** 0 ability to lift an extended knee at the hip 1 ability to flex the knee but not to lift an extended leg 2 ability to flex toes only 3 inability to move hip, knees, or toes	0 0 2 (7.4) 25 (92.6)	0 0 2 (7.4) 25 (92.6)	1.0

* Ramsay sedation scale

** Modified Bromage Scale

## Discussion

Spinal anesthesia is widely recognized as a safe and commonly employed technique for transurethral lithotripsy. Lower doses of intrathecal bupivacaine caused a reduction in the number of blocked dermatomes, and the duration of SA was reported. The co-administration of other adjuvant drugs can reduce the required bupivacaine dose and improve the quality of SA [3]. Several studies have reported improved surgical outcomes by including fentanyl, a lipophilic opioid, in spinal anesthesia [6, 14-20]. The study assessed the effects of fentanyl in co-administration with bupivacaine during SA, focusing on patient comfort, surgery success, the quality of the spinal block, and side effects.

While 74% of the study participants were male, no significant gender-based differences in statistical outcomes were observed between the two groups. However, the results suggest a potentially higher prevalence of kidney stones in men than expected. This discrepancy in the gender distribution of TUL candidates was also observed in a study by Uludağ*et al*. [21], in which 75% of patients were men. Although studies show that the prevalence of kidney stones in women has been increasing compared to the past [22], most patients with nephrolithiasis are men.

The sensory characteristics observed with bupivacaine alone or combined with intrathecal fentanyl revealed similar results. However, adding fentanyl to bupivacaine significantly increased SBL without increasing the recovery room discharge. The duration of the sensory block was 71.11±11.79 minutes in group A, but after adding fentanyl, this time increased to 86.11±17.77 minutes (group B) (p<0.001). Shahverdi *et al*. reported that among patients who underwent transurethral resection of the prostate (TURP), the mean total sensory block recovery time in the group of patients who received bupivacaine+fentanyl was significantly lower than the group of bupivacaine alone (66.2 *vs*. 77.7; p<0.001). However, the mean time to reach the sensory level was significantly longer in the bupivacaine + fentanyl group [20].

The overall mean duration of surgery was 49.44±14.46 minutes, and the two groups were not significantly different in this regard. This is similar to the mean operative time reported in a study by Uygulanan *et al*. [23], which was 44.2±20.4 minutes for transurethral lithotripsy (TUL) under spinal anesthesia. It was demonstrated that the time of onset of sensory block was about 3-5 minutes, and the two groups were not significantly different in terms of sensory block onset time and level of sensory block. Similar to our findings, no significant difference was seen between the study groups concerning the onset time and level of sensory block [18, 24]. Still, patients who received bupivacaine with fentanyl demonstrated a marked decrease in the necessity for intravenous analgesics during and after surgery [15, 16]. The patient's pain, relaxation degree, and the level of movement block were not different between groups.

Postoperative pain intensity, assessed by VAS score, was reported between 0 and 1 in both groups. Similarly, the level of sedation, as indicated by the RSS score, was consistently within the range of 1 to 2 in both groups. Additionally, the degree of motor block, assessed using the modified Bromage Score, was identical in both groups, with a score of 2.92±0.26. This score signifies that patients had limited or no movement in their lower limbs, including the pelvis, knees, and ankles, with a score of 3 indicating complete immobility and a score of 2 indicating limited ankle movement. These results are consistent with the findings of Atallah *et al*. [18], which found that patients receiving bupivacaine with or without fentanyl were not significantly different in terms of motor block, and deep motor block occurred in both groups. Since the ability to move in either group was not more remarkable than the ankle, it can be concluded that the use of bupivacaine alone or in combination with fentanyl can prevent the patient from moving by creating a safe level of temporary movement block during the surgery and the possibility of traumatic injuries following the patient's mobility is low.

The results of the present study confirmed that the addition of fentanyl to low-dose bupivacaine increased the success rate (stone-free rate) of TUL as complete stone-free status was achieved in 100% of the patients in group B, which was marginally insignificant (p=0.051). Many studies compared the stone-free rate in different anesthesia methods used in urologic surgeries [23, 25]. According to the findings of this study, the overall incidence of anesthesia-related complications ranged from 8% to 10%. The most commonly reported side effects were shivering, nausea, vomiting, and bradycardia, which were generally well tolerated. These findings confirm the results of the previous reports, in which the incidence of side effects was low and controllable [18, 24, 26]. Shivering after anesthesia is very common, and its higher prevalence in group A is due to a lack of opioids [27]. Vomiting and nausea are unavoidable complications in patients receiving opioids [28-30].

Some studies reported that the most common side-effect following intrathecal administration of fentanyl was pruritus [31, 32]. However, in this study, no patient had pruritus requiring treatment, consistent with previous investigations [17, 25, 33]. Furthermore, when examining the incidence of surgical complications in the two groups, none of the patients receiving the bupivacaine-fentanyl combination had stone retropulsion. The stone-free rate in this group was 100%. In comparison, the prevalence of this complication was reported to be up to 18.5% in the group receiving bupivacaine alone. In the study by Topaktaş*et al*. [34].in the initial month following surgery, the stone-free success rate was 90% (36 out of 40) in the SA group and 93.7% (30 out of 32) in the GA group. Their findings suggest that ureterorenoscopy is a dependable surgical approach for adult patients with proximal ureter stones. The procedure can be carried out successfully under either SA or GA anesthesia. Stone retropulsion can manifest due to various factors, including surgical technique, such as using high water pressure to access the stone, or patient-related factors, like the inability to use a basket due to ureteral inflammation, ureteral stricture, or an unstable patient condition. These factors can lead to discontinuation of surgery. In this study, surgical technique and patient factors were not assessed. Therefore, significant differences in retropulsion between the two groups may be multifactorial and cannot be explained by anesthetic factors alone.

The strength of this study lies in its pioneering approach to spinal anesthesia for TUL, demonstrating the safety of using intrathecal opium in SA. The main limitation of this study is being monocentric. Therefore, it is recommended that future research with larger sample sizes consider assessing surgical techniques and patient characteristics alongside the anesthetic approach to gain a more comprehensive understanding of the factors contributing to complications.

## CONCLUSION

This study demonstrated that using bupivacaine with or without fentanyl in spinal anesthesia provided a reliable sensory and motor block level in patients with urolithiasis who were candidates for TUL. However, the addition of intrathecal fentanyl was associated with a lower incidence of stone retropulsion. The factors affecting the occurrence of this complication and the effect of anesthetic drugs on the success rate of surgery should be considered.

## Personal thanks

We gratefully acknowledge the support provided by the Urology Research Center at Guilan University of Medical Sciences. We thank all urology staff of Razi Hospital, Rasht, for their assistance in the study.
